# Modelling Cyclic Behaviour of Martensitic Steel with J2 Plasticity and Crystal Plasticity

**DOI:** 10.3390/ma12111767

**Published:** 2019-05-31

**Authors:** Hafiz Muhammad Sajjad, Stefanie Hanke, Sedat Güler, Hamad ul Hassan, Alfons Fischer, Alexander Hartmaier

**Affiliations:** 1 Interdisciplinary Centre for Advanced Material Simulation (ICAMS), Ruhr-Universität Bochum, Universitätsstr 150, 44801 Bochum, Germany; hamad.ulhassan@rub.de (H.u.H.); Alexander.Hartmaier@icams.rub.de (A.H.); 2 Materials Science and Engineering, University of Duisburg Essen, Lotharstr. 1, 47057 Duisburg, Germany; stefanie.hanke@uni-due.de (S.H.); sedat.gueler@uni-due.de (S.G.); alfons.fischer@uni-due.de (A.F.)

**Keywords:** cyclic loading, kinematic hardening, crystal plasticity, homogenization, fatigue

## Abstract

In order to capture the stress-strain response of metallic materials under cyclic loading, it is necessary to consider the cyclic hardening behaviour in the constitutive model. Among different cyclic hardening approaches available in the literature, the Chaboche model proves to be very efficient and convenient to model the kinematic hardening and ratcheting behaviour of materials observed during cyclic loading. The purpose of this study is to determine the material parameters of the Chaboche kinematic hardening material model by using isotropic J2 plasticity and micromechanical crystal plasticity (CP) models as constitutive rules in finite element modelling. As model material, we chose a martensitic steel with a very fine microstructure. Thus, it is possible to compare the quality of description between the simpler J2 plasticity and more complex micromechanical material models. The quality of the results is rated based on the quantitative comparison between experimental and numerical stress-strain hysteresis curves for a rather wide range of loading amplitudes. It is seen that the ratcheting effect is captured well by both approaches. Furthermore, the results show that concerning macroscopic properties, J2 plasticity and CP are equally suited to describe cyclic plasticity. However, J2 plasticity is computationally less expensive whereas CP finite element analysis provides insight into local stresses and plastic strains on the microstructural length scale. With this study, we show that a consistent material description on the microstructural and the macroscopic scale is possible, which will enable future scale-bridging applications, by combining both constitutive rules within one single finite element model.

## 1. Introduction

Highly nitrogen-alloyed martensitic steels are suitable for many engineering applications (industrial cutting knifes, roller bearings in aerospace industry) due to their excellent corrosion resistance, enhanced yield strength, tensile strength and better low cycle fatigue (LCF) resistance in comparison to the typical austenitic stainless steels with a smaller amount of nitrogen [1,2,3,4]. These beneficial effects of nitrogen make these alloys a good candidate for engineering applications that demand corrosion resistance along with high mechanical strength. In recent years, many experimentalists have carried out studies in order to understand the LCF behaviour of ferritic-austenitic as well as fully austenitic [5,6] high nitrogen-alloyed steels [7,8,9]. Nevertheless, as fatigue experiments are time- and cost-intensive, it is of high interest to determine fatigue properties by use of efficient numerical simulations [10,11]. Such virtual experiments require a material model in the form of a constitutive rule to perform finite element simulations. Owing to a vast range of available material models for cyclic plasticity [12,13], selecting a suitable material model for the desired application is quite a challenging task. A brief historical overview of different material models with their characteristic features is presented in the following section.

The stress-strain response of metallic materials under cyclic loading is complicated, and thus, it is difficult to describe the mechanical behaviour of different materials with only one plasticity modelling approach. In order to cope with this problem, many different rate-independent plasticity models have been suggested in the literature. For example, Prager-Ziegler [14] proposed the simplest linear kinematic hardening rule in order to capture the Bauschinger effect [15]. Ziegler suggested that yield surface translates radially with respect to the centre. Mróz [16] and Iwan [17], working independently, have presented a multi-surface model assuming that cyclic non-linearity of metals depends on the loading histories. This model is capable of describing cyclic hardening or softening and the stress-strain hysteresis. In another study, Dafalias and Popov [18,19] and Krieg [20], independently tried to propose a two-surface model instead of using multi-surface for describing the nonlinearity of metallic materials. They described the yielding condition by yield surface and the limiting state of the stress was defined by bounding surface. The hardening moduli were replaced by a general plastic modulus in the two-surface model. These hardening models helped to understand the material response in a much better way, but, similar to the other multilinear models, they are also unable to predict ratcheting behaviour [21].

In order to study the nonlinearity of the material, Armstrong [22] modified the Prager linear kinematic hardening model and suggested a new approach to capture the cyclic behaviour of the material by introducing the relaxation term into the calculation of back-stress. One drawback of the Armstrong model [22] was the assumption of a constant strain increment in each cycle. Chaboche [23,24] has addressed this limitation in the Armstrong model and suggested decomposition of backstress (or summing law) into more than one term for nonlinear kinematic behaviour. The Chaboche model is actually a generalized form of the Armstrong model for describing nonlinear plasticity in different materials at various strain ranges and is available in most of the well-known commercial software-packages. In order to achieve a better ratcheting prediction for cyclic nonlinear kinematic hardening, many evolution laws based on the Chaboche model [25,26,27] are available in the literature and Chaboche himself has comprehensively summarised well-known plasticity models in his work [28].

The Chaboche model can deal with macroscopic and micromechanical problems in order to identify material parameters using the inverse technique. Liu et al. [29], used the Chaboche material model for optimising material parameters and concluded that initial yield stress plays an important role in this optimisation and recommended to ignore the elastic part from the experimental stress-strain curve for the optimisation. In another study, Bari and Hassan [30] compared the performance of five different constitutive models based on their ability to simulate the ratcheting effect and concluded that the Chaboche model performed quite well in capturing the uniaxial ratcheting response. In a recent study, Schaefer et al. [31] showed the limitation of Chaboche model in predicting mean stress relaxation behaviour at a different total strain ratio.

Moeini et al. [32,33] used the Chaboche material model to predict the micromechanical response in dual phase (DP) steels and identified material parameters with three back stress terms leading to the conclusion that strain accumulates in soft ferrite. They further used a 2D representative volume element (RVE) to predict the LCF behaviour of DP steels using micromechanical modelling. Boeff et al. [34] have presented a micromechanical based model to study fatigue crack initiation of polycrystalline materials and also showed the effect of hardening and softening mechanisms on the evolution of stresses in the microstructure.

Literature that compares both the aforementioned (macro and micromechanical) approaches for Chaboche material parameters in low cycle fatigue tests [35] is, however, rare. In the present study, an effort has been made to compare both approaches for material parameter identification by using the Chaboche kinematic hardening model at the macroscopic and the micromechanical level. The aim of this study is to investigate, whether the Chaboche kinematic hardening model [23,24] allows us to give a consistent material description on both scales, which in turn would enable future applications within scale-bridging materials modelling.

## 2. Materials and Experiments

**Figure 1:**
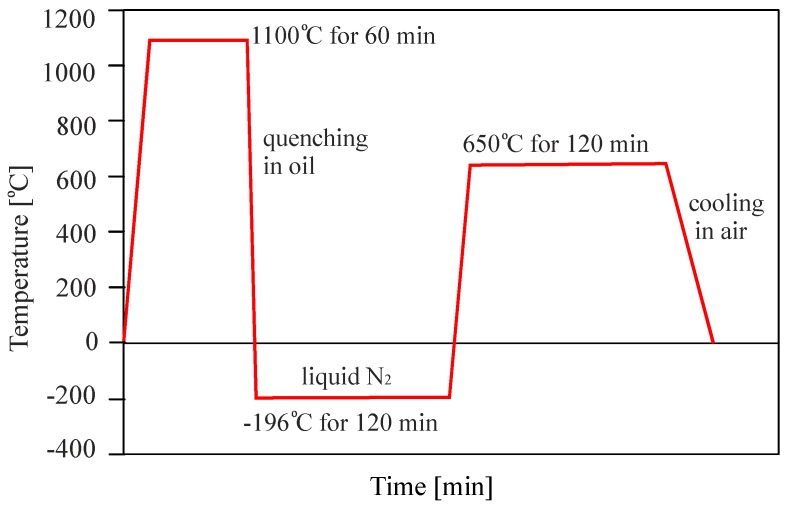
Heat treatment procedure.

The material employed for this study is the martensitic high-nitrogen stainless steel X30CrMoN15-1 (1.4108; AMS 5898, Energietechnik Essen GmbH, Essen, Germany), typically used for engineering applications. The chemical composition, as provided by the manufacturer, is given in Table 1. Bar stock material was used, and bars of 20 mm diameter were cut into sections of 100 mm length. These were heat-treated in order to achieve a hardened and tempered state with little or no retained austenite. The material was first hardened and tempered by heating and holding in the austenite regime at 1100 °C for 60 min, followed by quenching in oil and deep cooling in liquid nitrogen at −196 °C for 120 min. Following the cryogenic treatment, the bars were tempered at 650 °C for 120 min and left to cool in air. The heat treatment is summarized in the schematic in Figure 1. The hardness obtained through this treatment, measured by Vickers’ method, is 385 ± 9 HV10 (Hardness according to Vickers with test load of 10 kg).

**Figure 2:**
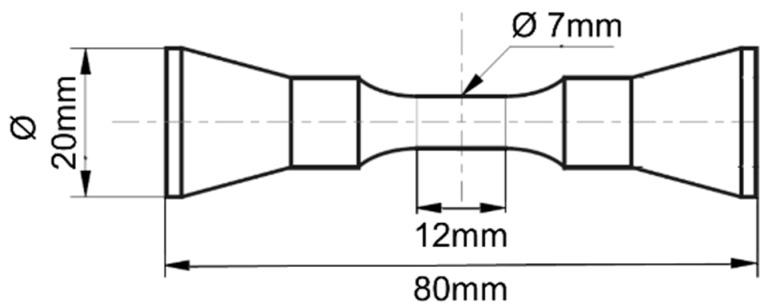
Specimen geometry for fatigue testing.

Following the heat treatment, the bars were machined into dog bone shaped, cylindrical specimen according to the sketch in Figure 2. The surface of the cylindrical gauge length was mechanically polished using diamond paste down to 1 µm grain size.

Uniaxial fatigue experiments were conducted on a servo-hydraulic testing machine (Bionix 858, MTS, Eden Prairie, MN, USA) with a possible maximum load in tension and compression of 50 kN. The specimen was clamped form-fitted on the cone-shaped ends for applying tensile forces. Compressive forces were applied via a punch on the face of the specimen. An extensometer (632.13F-20, MTS, Eden Prairie, MN, USA) was placed on the gauge length to control the strain.

Experiments were carried out strain-controlled, in air and at room temperature. Each experiment was conducted at a specific chosen total-strain amplitude ($\varepsilon_{a,t}$) with a value of *R*_ε_ = −1 (ratio of lower to upper strain). Test frequencies of 0.5 Hz were chosen, in order to avoid heating of the specimen due to energy dissipation. Experiments were run up to specimen failure or to 2 × 10^6^ cycles. Strain, axial force, time and the number of cycles were recorded at a frequency of about 2 kHz.

Metallographic characterization of the steel was carried out, and samples were prepared according to standard methods up to a mechanical polishing using diamond suspension of 1 µm grain size. For light microscopic analysis, samples were etched using a solution containing 33 mL H_2_O, 33 mL ethanol, 33 mL HCl and 1.5 g CuCl_2_ (“Kalling 1”). Scanning electron microscopy (SEM) and electron backscatter diffraction (EBSD) was employed to further analyze the microstructure and determine the content of retained austenite. For EBSD, an additional vibratory polishing (VibroMet 2, Buehler/ITW Test & Measurement GmbH, Düsseldorf, Germany) using 0.05 µm Silica suspension was applied for 3 h. An SEM with field emission gun (Leo 1530 Gemini, Carl Zeiss Microscopy GmbH, Jena, Germany) was used, equipped with an EBSD system (Digiview IV CCD camera, EDAX Inc., Mahwah, NJ, USA).

**Figure 3:**
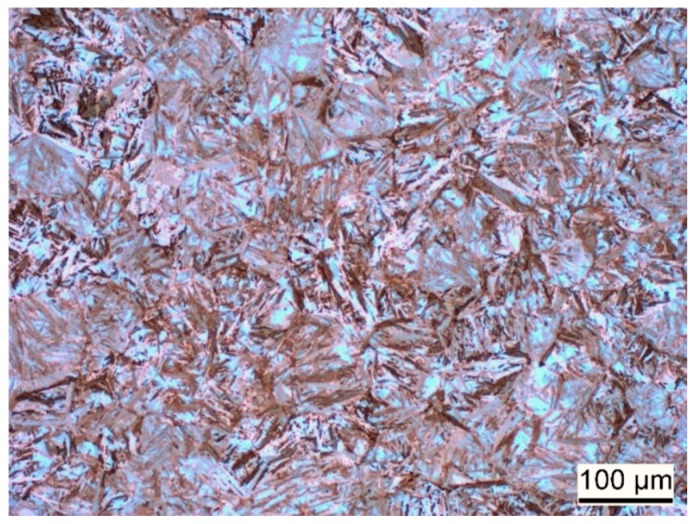
Light-microscopic image of etched sample, showing martensitic microstructure of the hardened and tempered steel.

**Figure 4:**
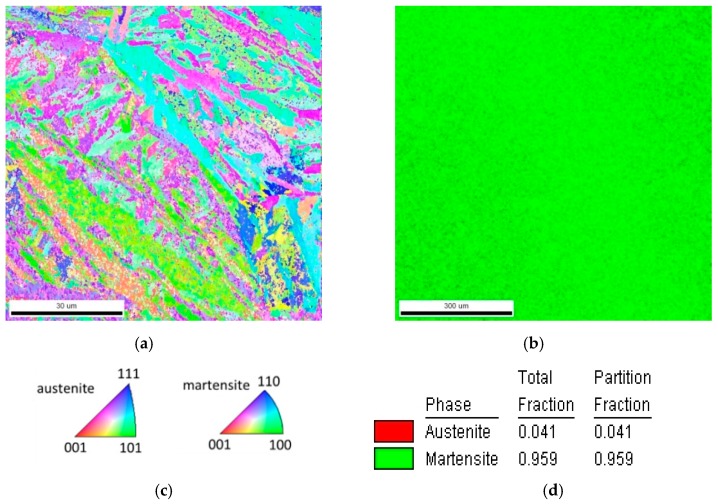
(**a**) EBSD map of the hardened and tempered steel.; (**b**) Phase map indicating ≈ 95% martensite in the microstructure. (**c**) Inverse pole figure for map in (**a**); (**d**) legend for phase fraction in (**b**).

The microstructure of the hardened and tempered steel is shown in Figure 3 and Figure 4. In both figures, martensite plates of varying size can be observed. The EBSD phase map in Figure 4b indicates a martensite phase fraction of ≈95%. It must be considered that the employed method may involve some error in the phase fraction, in particular, if very fine isles of retained austenite are present. Nevertheless, it can be concluded that the content of retained austenite is very low.

## 3. Modelling Methodology and Constitutive Model Optimization

Two separate numerical models are developed for the experimentally investigated martensitic steel X30CrMoN15-1. Section 3.1 describes the numerical models used for the present investigation. The Constitutive Chaboche model used for J2 plasticity and for CP is described in Section 3.2. Lastly, Section 3.3 explains the strategy of calibrating the material parameters.

### 3.1. Numerical Model

For simulating J2 plasticity, a 2D CPE4 (Four-node plane strain element with full integration) one-element model is considered sufficient [36], while in the case of CP, a 3D representative volume element (RVE) with 64 grains is used. Each grain is meshed into further 8 cubic C3D8 elements (with full integration) in order to reduce the influence of numerical stiffness at the grain boundaries. Segurado [37] showed that using only one cubic element per grain in FEM analysis shows stiffer behaviour under tensile loading and fails to predict texture evolution. Note, that we do not attempt to mimic the complex martensitic microstructure in this work, because in Schaefer et al. [31] it has been shown that a simple model with cubic grains is sufficient to describe macroscopic properties with good precision. If, however, local stresses and strains on the microstructural scale need to be analysed, a proper geometrical description of the grain shapes is mandatory, which in turn makes the numerical simulation much more demanding in terms of computer time. Hence, we decided to use the numerically efficient model here, because we only focus on the comparison of macroscopic stresses and strains.

**Figure 5:**
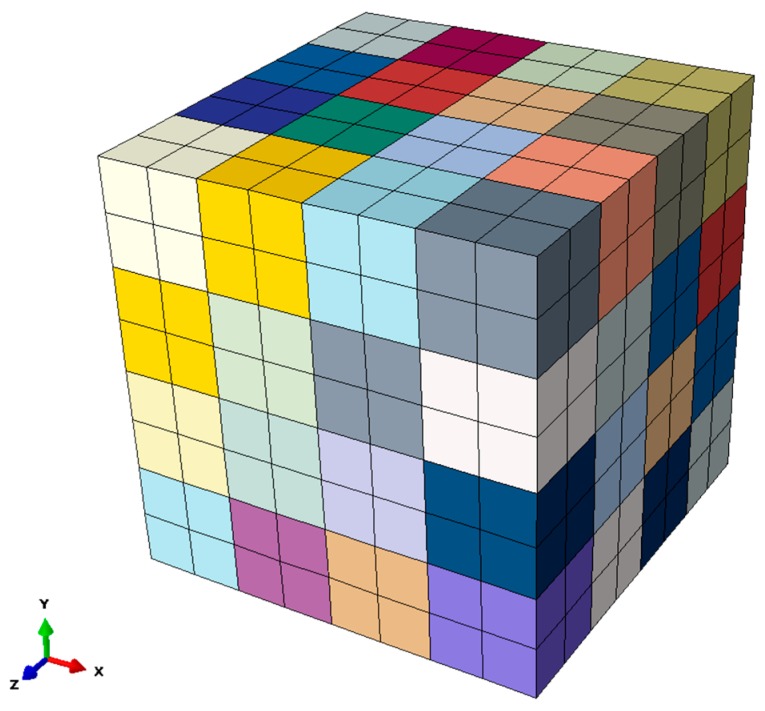
Finite element model used for crystal plasticity (CP).

Each grain of this model is assigned a random orientation, which is shown by using different colours in Figure 5. In this study, a homogenization scheme is used for extracting global stress. The purpose of using RVE for material simulation is to achieve the best results that will be difficult to achieve without applying periodic boundary conditions.

In case of Dirichlet boundary, the system behaves too rigid due to unrealistically high forces whereas Neumann boundary conditions allow the system to respond softly due to underconstrained deformation at the interface [38,39]. Displacement controlled loading is applied by using periodic boundary conditions for both models according to the work done by [40].

### 3.2. Material Model

In the following section, the Chaboche material model is explained in detail for J2 plasticity and for crystal plasticity.

#### 3.2.1. J2/Von Mises plasticity

The von Mises yield function explains that yielding starts when the second deviatoric stress invariant J2 reaches a critical value. The general form of this yielding criterion *f* is defined as

(1) $$f=\sqrt{\frac{3}{2}\left(S-\kappa \right):\left(S-\kappa \right)}\;-\left(\sigma_{0}+R\right),$$

where *S*, κ, $\sigma_{0}$ and *R* represents deviatoric stress, backstress, initial yield stress and isotropic hardening (i.e., the uniform expansion of yield surface) respectively [41]. In this study, the associative flow rule (i.e., plastic strain increment is proportional to the gradient of yield surface) for metals is used.

Hardening models are usually composed of isotropic hardening, kinematic hardening (i.e., yield surface translate in stress space) or a combination of these two models. The common form of isotropic hardening is

(2) $$R=Q\left(1-e^{-b\varepsilon_{eq}}\right),$$

where *Q* is the maximum change in the size of the yield surface,$\;\varepsilon_{eq}$ is the equivalent plastic strain and *b* determines the rate of isotropic hardening [42].

Here, the Chaboche material model [43] is used for modelling the combined, i.e., isotropic and kinematic hardening behaviour of the material. The kinematic hardening in the Chaboche material model is actually the extension of the Armstrong Frederick [44] model in which the only one back stress term (κ) is used, while in the Chaboche material model several backstress terms are used. This material model also has the capability to capture the ratcheting behaviour of the material under cyclic loading [28].

In the following equation, the kinematic hardening is described by the Chaboche material model, as

(3) $$\kappa =\overset{N}{\underset{i}{\sum }}\kappa_{i};\;d\kappa_{i}=\;\frac{2}{3}C_{i}d\varepsilon_{p}-g_{i}\kappa_{i}d\varepsilon_{eq}$$

**Figure 6:**
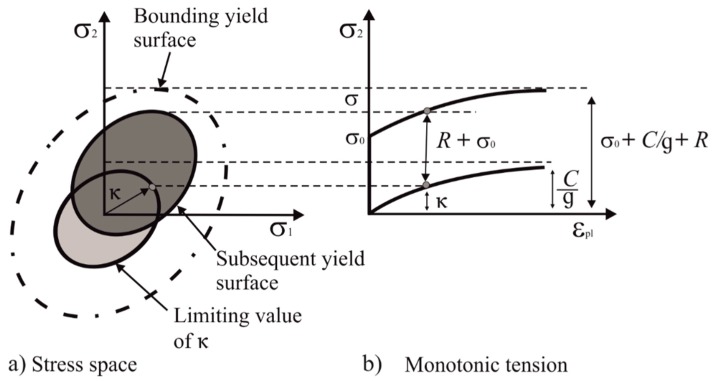
Graphical representation of the combined hardening evolution in the (**a**) stress space and (**b**) under monotonic tension after [42].

*C_i_* represents the kinematic hardening moduli while g_*i*_ describes the decrement rate of the related modulus with respect to plastic strain $d\varepsilon_{p}$. Figure 6 shows the graphical representation of the combined hardening evolution under monotonic tension and in the stress space [41].

As the Chaboche material model with two kinematic hardening terms (four unknown parameters) and isotropic softening (two unknown parameters) is used in the present study, six parameters are unknown; therefore, an inverse modelling technique for the determination of these parameters is applied.

#### 3.2.2. Crystal Plasticity (CP)

Crystalline materials exhibit a pronounced mechanical anisotropy in their elastic-plastic behaviour, which means that the mechanical response of crystalline materials depends on the loading path [12]. Crystal plasticity (CP) describes the relationship between the microstructural features like slip systems, dislocations, grain orientation and size, and the mechanical response of materials through mathematical constitutive formulation. It has its main applications in modelling material anisotropy, strain hardening, texture evolution, and damage, considering crystallographic orientations when deformation occurs in metals [12,45]. Crystal plasticity enables us to deal with the complicated internal/external boundary conditions imposed by inter- and/or intra-granular micro-mechanical interactions [46] in a computationally efficient manner.

In phenomenological crystal plasticity, critical resolved shear stress (CRSS) $\tau_{c}$ is the state parameter. CRSS is necessary to trigger the slip in grains, and thus it controls the plastic deformation. This constitutive model is implemented into the finite element solver ABAQUS 6.12 using a user-defined material subroutine (UMAT).

To calculate the shear rate, a prominent constitutive flow rule is described by

(4) $${\dot{\gamma }}^{a}={\dot{\gamma }}_{0}{\left|\frac{\tau^{a}-\chi_{bk}^{a}}{\tau_{c}^{a}}\right|}^{m}\mathrm{sign}\left(\tau^{a}-\chi_{bk}^{a}\right),$$

where $\dot{\gamma_{0}}$ and m are material parameters representing the reference shear rate and the shear rate sensitivity of the slip respectively.$\;{\dot{\gamma }}^{a}$ represents the shear rate with respect to the slip system a, and $\chi_{bk}^{a}$ gives resolved backstress on the same glide system a. This relationship is presented by Hutchinson, Peirce and Rice [47,48,49,50] in their work for metallic crystals. In order to capture isotropic hardening, commonly used work hardening rate is

(5) $${\dot{\tau }}_{c,\;iso}^{a}=\;\overset{N_{s}}{\underset{\beta =1}{\sum }}h_{0}{\left(1-\frac{\tau_{c}^{a}}{\tau_{c,s}}\right)}^{n}M_{\alpha \beta }\left|{\dot{\gamma }}^{\beta }\right|,\;$$

where $h_{0}$, n are the hardening parameters. $\tau_{c,s}$ represents the saturated CRSS and the evolution of CRSS between different slip systems are taken into account by the hardening matrix $M_{\alpha \beta }$. The backstress term in the Armstrong-Frederik model is defined as

(6) $$\dot{\chi_{bk}^{a}}=A{\dot{\gamma }}^{a}-B\chi_{bk}^{a}\left|{\dot{\gamma }}^{a}\right|$$

where A and B represent material parameters. Chaboche has generalized this backstress term in his model [23] which can be formulated in micromechanical modelling as

(7) $$\dot{\chi_{bk,\dot{i}}^{a}}=A_{i}{\dot{\gamma }}^{a}-B_{i}\chi_{bk}^{a}|{\dot{\gamma }}^{a}|,\;\chi_{bk}^{\;a}=\sum_{i=1}^{N_{B}}\chi_{bk}^{\;a}{,}_{i}$$

where *A* and *B* represent material parameters while *N*_B_ is the superposition of Armstrong-Frederik model. A brief introduction of some well-known plasticity models for cyclic loading has been reviewed above. The detailed discussion has just been presented for the Chaboche combined hardening model with J2 plasticity and crystal plasticity. In the next section, material parameters will be identified by using these two approaches and will be compared with the experimental results.

### 3.3. Optimization Algorithm

**Figure 7:**
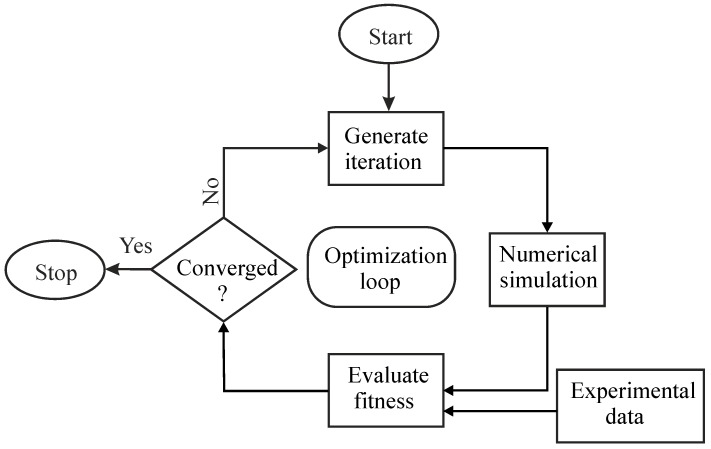
Optimisation loop used to optimise material parameters for J2 plasticity and CP.

The commercially available optimizer LS-Opt is used to identify the material parameters of the Chaboche model from the stress-strain hysteresis using inverse identification technique. The general optimization process which is adopted in determining Chaboche material parameters for J2 plasticity (defined in Equations (2) and (3)) and crystal plasticity (implemented using UMAT) is shown in Figure 7.

**Figure 8:**
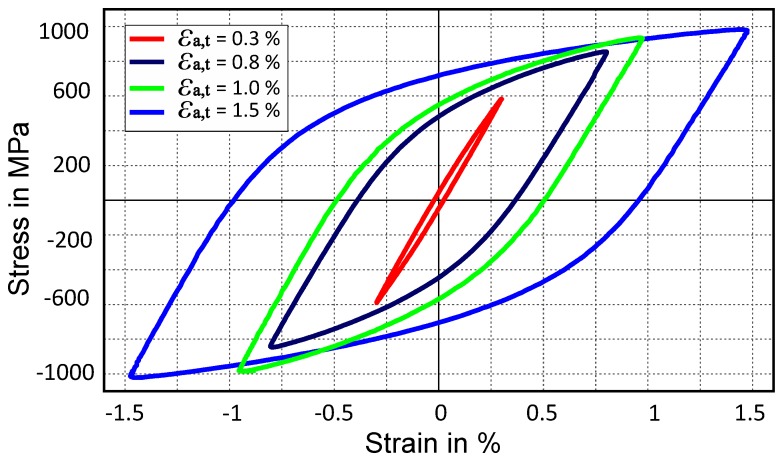
Experimental stabilized stress-strain hysteresis loops at half normalized lifetime for different total-strain amplitudes.

A curve mapping algorithm presented by ALT et al. [51] is used to capture the stress-strain hysteresis in this work [52]. The curve-mapping algorithm tries to minimize the area between the given curve (i.e., experimental curve) and the curve obtained from ABAQUS simulation. The stabilized cyclic stress-strain curves at total strain amplitudes of 0.30%, 0.80%, 1.00% and 1.50% as shown in Figure 8, are used as objectives for calibrating the Chaboche mode using J2 plasticity and CP. As more than one objective is needed to be optimized at the same time, a multi-objective strategy is deployed. In order to determine material parameters meeting all objectives (i.e., four experimental curves) at the same time, Pareto dominance criterion is used as it has the capability to consider multiple objectives simultaneously [53]. This multi-objective optimisation problem is solved using a genetic algorithm (GA) [54,55].

For every identification process, the genetic algorithm produces 100 generations where each generation consists of 30 simulations. After creating the first generation, it runs the 30 numerical simulations using a coupled FEM ABAQUS solver with Ls-Opt. The stress-strain hysteresis from the simulation is extracted by using a post-processing script written in Python. When one generation completes its 30 simulations, the optimizer evaluates the fitness error for each simulation with the respective experimental stress-strain hysteresis (target curve in LS-Opt) and compares it with the convergence criteria (error between experimental and simulation curve).

## 4. Results and Discussion

Figure 8 shows the stress-strain hysteresis for the chosen material for different total-strain amplitudes: 0.3% 0.8%, 1.0%, and 1.5%. Each stress-strain curve represents the saturated hysteresis at the respective total-strain amplitude. These experiments are performed under a strain controlled condition at room temperature.

**Figure 9:**
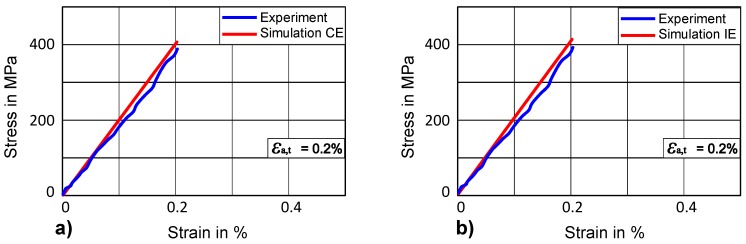
Comparison of stress-strain curve for elastic range (**a**) with crystal elasticity (CE) (**b**) with isotropic elasticity (IE).

First of all, the anisotropic elastic constants (C_11_, C_12_, C_44_) are determined with crystal elasticity. Elastic constants given by Kim and Johnson [56] are used as the starting point and values of these parameters are varied by 20% in order to identify the parameters which give the best fit for an experimental stress-strain curve up to a range of 0.2% total-strain amplitude. The optimized curve and the experimental curve for elastic regions show a quite good agreement, as can be seen in Figure 9a.

In case of isotropic elasticity, Young’s modulus is determined as 206 GPa taken from the experimental tensile stress-strain curve and validated by the simulation, where an acceptable match with the experimental curve can be seen in Figure 9b.

**Figure 10:**
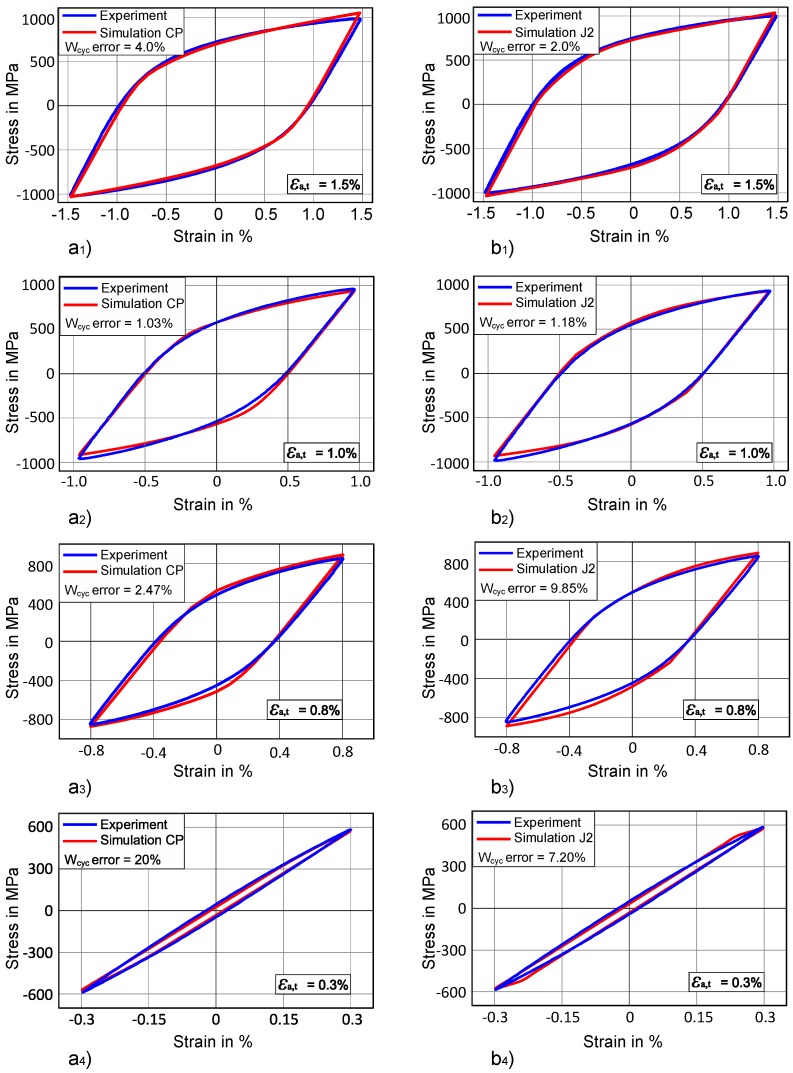
Optimised stress-strain hysteresis for CP (left side) and for J2 plasticity (right side) at total-strain amplitude of (**a_1_**,**b_1_**) 1.5%, (**a_2_**,**b_2_**) 1.0%, (**a_3_**,**b_3_**) 0.8%, and (**a_4_**,**b_4_**) 0.3% respectively.

Kinematic hardening parameters for the Chaboche model (Equation (3)) are identified in the next step. Saturated stress-strain hysteresis curves for four different total-strain amplitudes (i.e., 0.3%, 0.8%, 1.0%, 1.5%) are used from experiments. In order to identify the parameters, a multi-objective optimization is performed considering all four curves as a target, simultaneously. The parameters are identified for J2 plasticity and crystal plasticity. Isotropic hardening/softening parameters are kept zero during optimization of kinematic hardening parameters. This assumption is necessary because, at the saturation level for stress-strain hysteresis, isotropic hardening/softening parameters have no influence. Only two backstress terms (one linear and one nonlinear) are used for predicting hysteresis, which gave a good fit as shown in Figure 10 for J2 plasticity and crystal plasticity.

The stress-strain hystereses for optimized parameters at different total-strain amplitudes are shown in Figure 10. The blue line represents the experimental curve while the simulation curve is red. For a 1.5% total-strain amplitude, the simulated stress-strain hysteresis and the experimental hysteresis have shown a quite good match not only for CP but also for J2 plasticity as shown in Figure 10 (a_1_ and b_1_). When we compare the cyclic work *W*_cyc_ (i.e., dissipated strain energy) of the optimized curve with the experimental cyclic work at 1.5% strain, the difference is under 5%. CP has 4% error for *W*_cyc_ while in case of J2 this difference is only 2% as compared to the experimental *W*_cyc_.

In the case of 1.0% strain shown in Figure 10a_2_,b_2_, the prediction of the experimental curve is even better not only for curve matching but also for *W*_cyc_ in J2 as well as in CP. In this case, the error of simulated *W*_cyc_ as compared to experimental dissipated strain energy is only about 1% for both CP and J2 optimized parameters.

When we compare the optimized simulation results at 0.8% total-strain amplitude as shown in Figure 10a_3_,b_3_), CP shows a slightly better fit with the experimental curve while the J2 optimized stress-strain hysteresis shows some deviation from the experimental curve. The same trend can be observed in the calculation of *W*_cyc_ for both models. CP has about 2.5% *W*_cyc_ error while J2 plasticity has shown about 10% *W*_cyc_ difference as compared to the experimental *W*_cyc_.

In the case of a 0.3% total-strain amplitude, optimized stress-strain hysteresis proves to be a good qualitative match as compared to the experimental curve for both CP and J2 plasticity (Figure 10a_4_,b_4_). The J2 plasticity has about 7% error while CP has about 20% error for *W*_cyc_. Schaefer et al. [31] also have discussed the problem of getting a higher deviation from experimental data at lower total-strain amplitudes. This higher error at lower total-strain amplitude for both CP and J2 could be due to very low *W*_cyc_ in experiments at 0.3% strain, which makes the error calculation very sensitive. In addition to this, the algorithm tries to match the curve, which seems to be in good agreement (Figure 10a_4_,b_4_). In summarising all the above results, one can see that both approaches are able to capture cyclic behaviour of the material at different total-strain amplitude. The error deviation between simulation and experimental cyclic work *W*_cyc_ is in the range of 1% to 20% and this error increases with decreasing total strain amplitude. The optimized parameters for both crystal plasticity and J2 plasticity are shown in Table 2 and Table 3 respectively.

**Figure 11:**
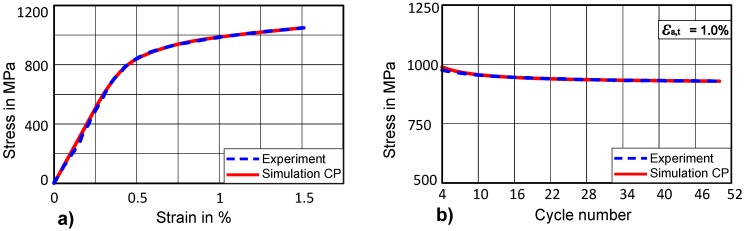
(**a**) Optimised uniaxial stress-strain curve for CP at ε_a,t_ = 1.5%; (**b**) Evolution of stress amplitude for (CP) first 50 cycles

For isotropic softening material parameters of CP, experimental uniaxial stress-strain data at 1.5% total-strain amplitude is used as a target curve. The kinematic hardening parameters already identified from a saturated cycle will be used as constants and they are not varied while optimizing isotropic parameters for CP. The result obtained after optimization shows a good fit for a given total-strain amplitude of 1.5% as shown in Figure 11a. After successfully determining isotropic softening material parameters from uniaxial stress-strain data, cyclic isotropic softening parameters are identified in the next step.

In order to identify cyclic isotropic softening parameters in CP, saturated CRSS (*τ*_c,s_) will have a lower value than the initial CRSS (*τ*_c,0_) due to the isotropic softening. In a study by Mahmoudi [55], it is shown that accurate hysteresis loop prediction is not enough to predict the ratcheting behaviour of the material. Therefore, the target curve is the evolution of maximum stress over the number of cycles for the first 50 cycles. The values of optimized isotropic softening parameters can be found in Table 2.

In the case of J2 plasticity, the Chaboche model implemented in ABAQUS has been applied to simulate cyclic isotropic hardening or softening behaviour. The martensitic steel used in this study shows isotropic softening over the number of cycles, therefore isotropic softening material parameters (Equation (2)) have been identified.

**Figure 12:**
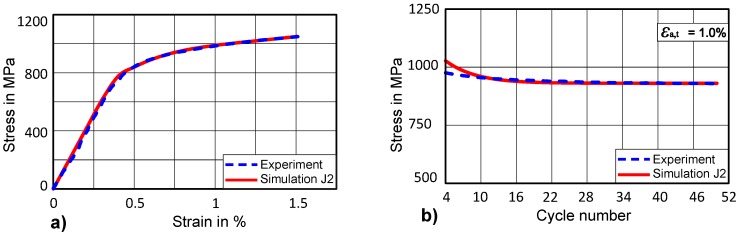
(**a**) Optimised uniaxial stress-strain curve for J2 at ε_a,t_ = 1.5% (**b**) Evolution of stress amplitude for (J2) first 50 cycles.

Firstly, uniaxial stress-strain is fitted for 1.5% strain as it has been done in case of CP as shown in Figure 12a. In order to determine cyclic isotropic softening material parameters for J2 plasticity, the same target curve is used representing the evolution of maximum stress over the number of the cycles for the first 50 cycles (Figure 12b). The optimized parameters of isotropic softening for J2 plasticity are given in Table 3. The comparison of the J2 optimized curve with the experimental curve is shown in Figure 12b.

By comparing the uniaxial stress-strain curve for J2 plasticity and CP, it can be observed that a good match with the experimental curve can be achieved as shown in Figure 11a and Figure 12a. However, analysing cyclic isotropic softening curves for J2 and CP, one could distinguish a difference in the quality of both fits (Figure 11b, Figure 12b). This difference is particularly visible in the initial 10 cycles, where CP has a better ability to capture both the cyclic behaviour of a material in the beginning as well as at the higher number of cycles. The main reason could be that the hardening models for CP are implemented in a user-defined fashion, which allows for the flexibility of coupled-decoupled evolution of isotropic hardening/softening and kinematic hardening.

## 5. Conclusions

In the present study, two different constitutive rules, i.e., J2 plasticity and crystal plasticity (CP), have been applied for studying the cyclic behaviour of martensitic steel. The differences between the models and their formulation has been given and it is noted that J2 plasticity is numerically much more efficient and thus is typically applied in macroscopic finite element simulations on the component level, whereas CP is applied in micromechanical models, where mechanical behaviour on the level of individual grains of a microstructure is considered. Cyclic stress-strain data from experiments at different total-strain amplitudes were used in order to identify all material parameters, including elastic constants and the parameters for isotropic and kinematic hardening. In particular, we fully parameterized the Chaboche kinematic hardening model for both constitutive rules by fitting simulated stress-strain hysteresis curves to the experimental data. Both plasticity models were able to capture cyclic plasticity for a considerable range of strain amplitudes with a constant parameter set for each constitutive rule. The deviation measured between the modelled and experimental hysteresis curve was in the range of 1% to 20%; where the larger error occurred for smaller strain amplitudes.

Furthermore, cyclic isotropic softening parameters were also identified with the help of both approaches. These parameters have been used to predict and to compare the uniaxial stress-strain curves. Here, CP showed a better ability to capture cyclic behaviour of material even in the first 10 cycles while J2 plasticity takes more cycles to capture the cyclic behaviour. In all cases, we found that the first 50 cycles are sufficient to capture cyclic plasticity for the purpose of fitting cyclic material parameters.

By showing that J2 plasticity and micromechanical CP models can be parameterized in a way to consistently describe real material behaviour, we open a way for future applications that use both constitutive rules simultaneously in scale-bridging finite element models, where regions that explicitly consider the microstructure, can be embedded in regions, where a more efficient constitutive rule is applied on a coarser mesh.

## Figures and Tables

**Table 1:** Chemical composition as provided by the manufacturer.

Element	Cr	Mo	Si	Mn	N	C	Ni	P	Al	V	Ti	Cu	S
wt.%	15.3	1.0	0.7	0.4	0.4	0.3	0.2	0.02	0.01	0.03	0.003	0.05	0.001

**Table 2:** Material parameters for the crystal plasticity model, including elastic constants and parameters for cyclic hardening.

Elastic Constants		Kinematic Hardening Parameters		Isotropic Hardening Parameters	
*C*_11_ (GPa)	263.001	*A*_1_ (MPa)	9598	$\dot{\gamma }$_0_ (s^−1^)	0.001
*C*_12_ (GPa)	111.693	*A*_2_ (MPa)	287609	*h*_0_ (MPa)	3800
*C*_44_ (GPa)	78.939	*B*_1_	3128	*τ*_c,0_ (MPa)	260.301
		*B*_2_	0	*τ*_c,s_ (MPa)	204.172
				*n*	1.998

**Table 3:** Material parameters for J2 plasticity, including isotropic elastic constants and parameter for cyclic hardening.

Elastic Constants		Kinematic Hardening Parameters		Isotropic Hardening Parameters	
*E* (GPa)	206	*C*_1_ (MPa)	130764	$\sigma_{0}$ (MPa)	754.2
v	0.3	*C*_2_ (MPa)	21892	*Q* (MPa)	−199.9
		g_1_	478	*b*	10.4
		g_2_	0		

## Nomenclature

LCF	Low cycle fatigue
2D	Two-dimensional
3D	Three-dimensional
RVE	Representative volume element
*S*	Deviatoric stress
κ	Backstress
$\sigma_{0}$	Initial yield stress
*R*	Isotropic hardening
*b*	Rate of isotropic hardening
*Q*	Maximum variation in the size of yield surface
$\varepsilon_{\mathrm{eq}}$	Equivalent plastic strain
$\varepsilon_{a,t}$	Total-strain amplitude
*C_i_*	Kinematic hardening moduli for J2
g_*i*_	Rate of kinematic hardening moduli for J2
*N*	Number of backstress terms for J2
*A_i_*	Kinematic hardening moduli for CP
*B_i_*	Rate of kinematic hardening moduli for CP
*N_B_*	Number of backstress terms for CP
$d\varepsilon_{p}$	Plastic strain
CP	Crystal plasticity
$\tau^{a}$	Resolved shear stress
CRSS ($\tau_{c}^{a}$)	Critical resolved shear stress
UMAT	User material subroutine
*m*	Rate sensitivity of the slip
$\dot{\gamma_{0}}$	Reference shear rate
DP	Dual phase
${\dot{\gamma }}^{a}$	Shear rate with respect to slip system a
$\chi_{bk}^{a}$	Resolved backstress on the glide system
$A_{1}$, $A_{2}$	Material parameters
(${\dot{\tau }}_{c,\;iso}^{a}$)	Evolution of CRSS
$h_{0}$, n	Slip hardening parameters
$\tau_{c,s}$	Saturated CRSS
$M_{\alpha \beta }$	Hardening matrix
FEM	Finite element method
*C*_11_, *C*_12_, *C*_44_	Elastic constants
*W*_cyc_	Dissipated strain energy
E	Young’s modulus
HV	Vickers’ hardness
CPE4	Four-node plane strain element

## References

[1] Reed R.P. (1989). Nitrogen in austenitic stainless steels. JOM.

[2] Berns H. (1996). Manufacture and Application of High Nitrogen Steels. ISIJ Int..

[3] Chang B., Zhang Z. (2012). Low cycle fatigue behavior of a high nitrogen austenitic stainless steel under uniaxial and non-proportional loadings based on the partition of hysteresis loops. Mater. Sci. Eng. A.

[4] Simmons J.W. (1996). Overview: High-nitrogen alloying of stainless steels. Mater. Sci. Eng. A.

[5] Schymura M., Fischer A. (2013). Metallurgical aspects on the fatigue of solution-annealed austenitic high interstitial steels. Int. J. Fatigue.

[6] Güler S., Schymura M., Fischer A., Droste M., Biermann H. (2018). The influence of the nitrogen/nickel-ratio on the cyclic behavior of austenitic high strength steels with twinning-induced plasticity and transformation-induced plasticity effects. Materwiss. Werkst..

[7] Degallaix S., Armas A.F., Marinelli M.C., Heren S. (2006). On the cyclic softening behavior of SAF 2507 duplex stainless steel. Acta Mater..

[8] Vogt J. (2001). Fatigue properties of high nitrogen steels. J. Mater. Process. Tech..

[9] Pola J. (2001). Analysis of the hysteresis loop in stainless steels II. Austenitic—ferritic duplex steel and the effect of nitrogen. Mater. Sci. Eng. A.

[10] Bru A. (2006). Numerical simulation of micro-crack initiation of martensitic steel under fatigue loading. Int. J. Fatigue.

[11] Gu C., Lian J., Bao Y., Xiao W., Münstermann S. (2019). Numerical Study of the Effect of Inclusions on the Residual Stress Distribution in High-Strength Martensitic Steels During Cooling. Appl. Sci..

[12] Roters F., Eisenlohr P., Hantcherli L., Tjahjanto D.D., Bieler T.R., Raabe D. (2010). Overview of constitutive laws, kinematics, homogenization and multiscale methods in crystal plasticity finite-element modeling: Theory, experiments, applications. Acta Mater..

[13] Bažant Z.P. (1992). Mechanics of Solid Materials.

[14] Ziegler H. (1959). A modification of Prager’s hardening rule. Quart. Appl. Math..

[15] Bauschinger J. Begründer der Mechanisch-Technischen Versuchsanstalten. https://www.hausarbeiten.de/document/132971.

[16] Mróz Z. (1967). On the description of anisotropic workhardening. J. Mech. Phys. Solids.

[17] Iwan W.D. (1967). On a Class of Models for the Yielding Behavior of Continuous and Composite Systems. J. Appl. Mech..

[18] Dafalias Y.F., Popov E.P. (1975). Ein Modell für Werkstoffe mit nichtlinearer Verfestigung unter zusammengesetzter Belastung. Acta Mech..

[19] Dafalias Y.F., Popov E.P. (1976). Plastic Internal Variables Formalism of Cyclic Plasticity. J. Appl. Mech..

[20] Krieg R.D. (1975). A Practical Two Surface Plasticity Theory. J. Appl. Mech..

[21] Rezaiee-Pajand M., Sinaie S. (2009). On the calibration of the Chaboche hardening model and a modified hardening rule for uniaxial ratcheting prediction. Int. J. Solids Struct..

[22] Armstrong P.J., Frederick C.O. (1966). A Mathematical Representation of the Multiaxial Bauschinger Effect.

[23] Chaboche J.L., Dang Van K., Cordier G. (1979). Modelization of the Strain Memory Effect on the Cyclic Hardening of 316 Stainless Steel.

[24] Chaboche J.L. (1986). Time-independent constitutive theories for cyclic plasticity. Int. J. Plast..

[25] Ohno N., Wang J.-D. (1993). Kinematic hardening rules with critical state of dynamic recovery, part I: Formulation and basic features for ratchetting behavior. Int. J. Plast..

[26] Mcdowell D.L. (1995). Stress state of cyclic ratchetting behavior of two rail steels introduced modifications of the AF rule to more accurately model ratchetting effects A. Int. J. Plast..

[27] Ohno N. (2000). Kinematic hardening model suitable for ratchetting with steady-state. Int. J. Plast..

[28] Chaboche J.L. (2008). A review of some plasticity and viscoplasticity constitutive theories. Int. J. Plast..

[29] Liu S., Liang G. (2017). Optimization of Chaboche kinematic hardening parameters by using an algebraic method based on integral equations. J. Mech. Mater. Struct..

[30] Bari S., Hassan T. (2000). Anatomy of coupled constitutive models for ratcheting simulation. Int. J. Plast..

[31] Schäfer B., Song X., Sonnweber-Ribic P., ul Hassan H., Hartmaier A. (2019). Micromechanical Modelling of the Cyclic Deformation Behavior of Martensitic SAE 4150—A Comparison of Different Kinematic Hardening Models. Metals.

[32] Moeini G., Ramazani A., Sundararaghavan V., Koenke C. (2017). Micromechanical modeling of fatigue behavior of DP steels. Mater. Sci. Eng. A.

[33] Moeini G., Ramazani A., Myslicki S., Sundararaghavan V., Könke C. (2017). Low Cycle Fatigue Behaviour of DP Steels: Micromechanical Modelling vs. Validation. Metals.

[34] Boeff M., Hassan H. ul, Hartmaier A. (2017). Micromechanical modeling of fatigue crack initiation in polycrystals. J. Mater. Res..

[35] Velay V., Bernhart G., Penazzi L. (2006). Cyclic behavior modeling of a tempered martensitic hot work tool steel. Int. J. Plast..

[36] Ridha Hambli A.P. (2007). Comparison between 2D and 3D numerical modeling of superplastic forming processes Ridha. Comput. Methods Appl. Mech. Eng..

[37] Segurado J., Llorca J. (2013). Simulation of the deformation of polycrystalline nanostructured Ti by computational homogenization. Comput. Mater. Sci..

[38] Kulosa M., Neumann M., Boeff M., Gaiselmann G., Schmidt V., Hartmaier A. (2017). A Study on Microstructural Parameters for the Characterization of Granular Porous Ceramics Using a Combination of Stochastic and Mechanical Modeling. Int. J. Appl. Mech..

[39] Boeff M. (2016). Micromechanical Modelling of Fatigue Crack Initiation and Growth. Ph.D. Thesis.

[40] Smit R.J.M., Brekelmans W.A.M., Meijer H.E.H. (1998). Prediction of the mechanical behavior of nonlinear heterogeneous systems by multi-level finite element modeling. Comput. Methods Appl. Mech. Eng..

[41] Novak J.S., Benasciutti D., De Bona F., Stanojević A., De Luca A., Raffaglio Y. (2016). Estimation of material parameters in nonlinear hardening plasticity models and strain life curves for CuAg alloy. IOP Conf. Ser. Mater. Sci. Eng..

[42] Lemaitre J., Chaboche J.-L. (1990). Mechanics of Solid Materials.

[43] Chaboche J.L. (1989). Constitutive equations for cyclic plasticity and cyclic viscoplasticity. Int. J. Plast..

[44] Frederick C.O., Armstrong P.J. (2007). A mathematical representation of the multiaxial Bauschinger effect. Mater. High Temp..

[45] Roters F., Eisenlohr P., Bieler T.R., Raabe D. (2010). Crystal Plasticity Finite Element Methods: In Materials Science and Engineering.

[46] Sachtleber M., Zhao Z., Raabe D. (2002). Experimental investigation of plastic grain interaction. Mater. Sci. Eng. A.

[47] Rice J.R. (1971). Inelastic constitutive relations for solids: An internal-variable theory and its application to metal plasticity. J. Mech. Phys. Solids.

[48] Peirce D., Asaro R.J., Needleman A. (1982). An analysis of nonuniform and localized deformation in ductile single crystals. Acta Metall..

[49] Peirce D., Asaro R.J., Needleman A. (1983). Material rate dependence and localized deformation in crystalline solids. Acta Metall..

[50] Hutchinson J. (1976). Bounds and self-consistent estimates for creep of polycrystalline materials. Proc. R. Soc. Lond. A Math. Phys. Eng. Sci..

[51] ALT H., GODAU M. (2004). Computing the Fréchet Distance Between Two Polygonal Curves. Int. J. Comput. Geom. Appl..

[52] Witowski K. Identification of Material Parameters with LS-OPT. https://www.dynamore.de/de/download/papers/2014-ls-dyna-forum/documents/workshops/identification-of-material-parameters-with-ls-opt-r.

[53] Zitzler E., Laumanns M., Thiele L. (2001). SPEA2: Improving the Strength Pareto Evolutionary Algorithm.

[54] Agius D., Kajtaz M., Kourousis K.I., Wallbrink C., Wang C.H., Hu W., Silva J. (2017). Sensitivity and optimisation of the Chaboche plasticity model parameters in strain-life fatigue predictions. Mater. Des..

[55] Mahmoudi A.H., Pezeshki-Najafabadi S.M., Badnava H. (2011). Parameter determination of Chaboche kinematic hardening model using a multi objective Genetic Algorithm. Comput. Mater. Sci..

[56] Kim S.A., Johnson W.L. (2007). Elastic constants and internal friction of martensitic steel, ferritic-pearlitic steel, and α-iron. Mater. Sci. Eng. A.

